# Identification of rifampin-regulated functional modules and related microRNAs in human hepatocytes based on the protein interaction network

**DOI:** 10.1186/s12864-016-2909-6

**Published:** 2016-08-22

**Authors:** Jin Li, Ying Wang, Lei Wang, Xuefeng Dai, Wang Cong, Weixing Feng, Chengzhen Xu, Yulin Deng, Yue Wang, Todd C. Skaar, Hong Liang, Yunlong Liu

**Affiliations:** 1 College of Automation, Harbin Engineering University, 145 Nantong Street, Nangang District, Harbin, Heilongjiang 150001 China; 2 Network Information Center, Qiqihar University, No.42, Wenhua Street, Qiqihar, Heilongjiang 161006 China; 3 Department of Medical and Molecular Genetics, Indiana University School of Medicine, Indianapolis, IN USA; 4 Division of Clinical Pharmacology, Department of Medicine, Indiana University School of Medicine, Indianapolis, IN USA; 5 Center for Computational Biology and Bioinformatics, Indiana University School of Medicine, Indianapolis, IN USA

## Abstract

**Background:**

In combination with gene expression profiles, the protein interaction network (PIN) constructs a dynamic network that includes multiple functional modules. Previous studies have demonstrated that rifampin can influence drug metabolism by regulating drug-metabolizing enzymes, transporters, and microRNAs (miRNAs). Rifampin induces gene expression, at least in part, by activating the pregnane X receptor (PXR), which induces gene expression; however, the impact of rifampin on global gene regulation has not been examined under the molecular network frameworks.

**Methods:**

In this study, we extracted rifampin-induced significant differentially expressed genes (SDG) based on the gene expression profile. By integrating the SDG and human protein interaction network (HPIN), we constructed the rifampin-regulated protein interaction network (RrPIN). Based on gene expression measurements, we extracted a subnetwork that showed enriched changes in molecular activity. Using the Kyoto Encyclopedia of Genes and Genomes (KEGG), we identified the crucial rifampin-regulated biological pathways and associated genes. In addition, genes targeted by miRNAs that were significantly differentially expressed in the miRNA expression profile were extracted based on the miRNA-gene prediction tools. The miRNA-regulated PIN was further constructed using associated genes and miRNAs. For each miRNA, we further evaluated the potential impact by the gene interaction network using pathway analysis.

**Results and Disccussion:**

We extracted the functional modules, which included 84 genes and 89 interactions, from the RrPIN, and identified 19 key rifampin-response genes that are associated with seven function pathways that include drug response and metabolism, and cancer pathways; many of the pathways were supported by previous studies. In addition, we identified that a set of 6 genes (*CAV1, CREBBP, SMAD3, TRAF2, KBKG, and THBS1*) functioning as gene hubs in the subnetworks that are regulated by rifampin. It is also suggested that 12 differentially expressed miRNAs were associated with 6 biological pathways.

**Conclusions:**

Our results suggest that rifampin contributes to changes in the expression of genes by regulating key molecules in the protein interaction networks. This study offers valuable insights into rifampin-induced biological mechanisms at the level of miRNAs, genes and proteins.

**Electronic supplementary material:**

The online version of this article (doi:10.1186/s12864-016-2909-6) contains supplementary material, which is available to authorized users.

## Background

Protein-protein interactions are intrinsic to most biological processes [1]. Expanded knowledge of the protein interaction network (PIN) may shed light on basic cellular mechanisms. An expression profile is a dynamic collection of data used to deduce a gene’s function, state, environment, etc. With the increasing availability of genome and proteome data, the PIN can be integrated with gene expression profiles to create conditional network modules within a specific biological state. This method has been used to explore cellular mechanisms associated with multiple diseases [2], including cancer. For instance, Zhang et al. [3] analysed the genes and crucial modules associated with coronary artery diseases (CAD), and suggested that two proteins were critical for the development of CAD. Lin et al. [2] studied dynamic functional modules and co-expressed protein interaction networks in cases of dilated cardiomyopathy. Previous studies suggest that the integrated analysis of PIN and gene expression profile information may contribute to the identification of the functional modules and key genes that are relevant to important biological pathways.

Rifampin is a drug that is usually used to treat tuberculosis and inactive meningitis [4]. The molecular mechanisms and functions of rifampin-regulation have previously been identified. Our previous study has confirmed that rifampin altered expression level of miRNAs and many cytochrome P450 enzymes (CYPs), which are the major metabolic enzymes that control the metabolism of most clinically important drugs, and some of the changes exist in associated relationships that suggest that some of CYP mRNAs are targeted by some miRNAs [5–8]. Rifampin is also a typical ligand of the pregnane X receptor (PXR), which is a transcription factor and a key regulator of the CYPs and other genes involved in drug disposition [9, 10]. Furthermore, rifampin can rapidly downregulate hepatic angiogenesis- and mitogenesis-related genes. Therefore, it shows favorable antiproliferative effects on endothelial cell, which is make it potentially beneficial for targeting hepatobiliary cancer cells [11, 12].

Previous studies have demonstrated that the drug-metabolizing enzymes [6], transporters, and microRNAs (miRNAs) are regulated by rifampin [11, 12], and the mechanisms of the regulation of some of these genes are well-studied; however, little has been done to put the global gene expression effects of rifampin into biological pathways and interactive networks. Protein interaction network can depict and integrate information pertaining to domain architecture, post-translational modification, interaction networks and disease association for each protein in the human proteome [13]. Furthermore, by combining with mRNA expression profiles, they can be used to identify specific correlations of between the genes, and to identify the key genes and functional modules associated with critical biological pathways. In addition, the integration of the miRNA expression profiles can depict relationship between the altered expression of miRNAs and their targeted-mRNA. The implementation of an integrative method that incorporates protein interaction networks and gene expression profiles to reveal conditional network modules associated with the rifampin-regulated biological processes becomes increasingly important in clarifying the regulatory mechanisms responsible for the rifampin effects on gene expression.

**Fig. 1:**
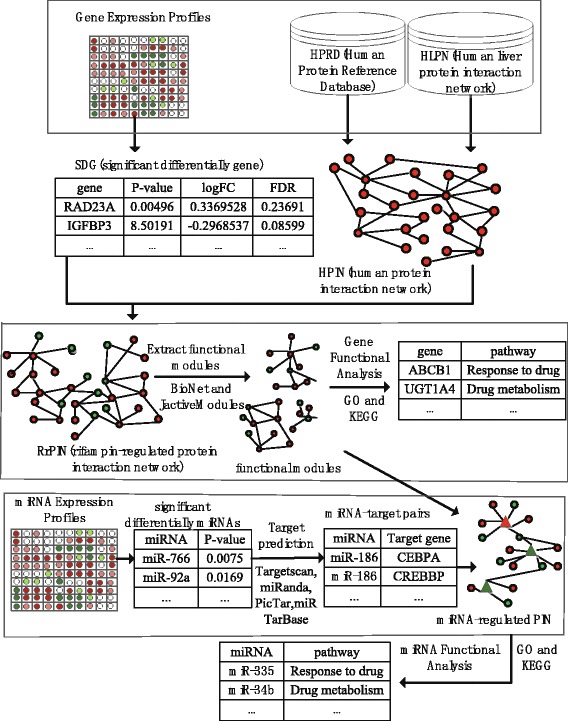
The hierarchical chart of identification of rifampin-regulated functional modules and related miRNAs

In this study, we focused on identifying the key genes, miRNAs, and the regulatory relationships among them. We also explored the rifampin-induced biological pathways by integrating the protein interaction networks and the miRNA and mRNA expression profiles. In this study, we propose a method which can be used to identify the rifampin-regulated functional modules in the protein interaction network of human hepatocytes, and can also be used to further analyze the rifampin-induced miRNAs and their functions. A schematic of the overall method is illustrated in Fig. 1. In this model, the gene expression profile and PIN are integrated to construct the rifampin-regulated protein interaction network (RrPIN). Then, in order to analyse the crucial biological pathways, we identify the functional modules that participate in a common biological function within the protein-protein interaction network. Next, the functional modules are extracted using BioNet and jActiveModules, and the rifampin-induced significant differentially expressed key genes are identified based on an analysis of Gene Ontology (GO) and the Kyoto Encyclopedia of Genes and Genomes (KEGG). Finally, the miRNA-regulated PINs are established using these key genes and gene-targeted miRNAs based on the miRNA expression profile and miRNA-target prediction databases, and the functions of the miRNAs are revealed based on GO and KEGG. The proposed analysis enables us to uncover rifampin-induced biological mechanisms in human hepatocytes.

## Methods

### Data

The gene expression dataset and miRNA expression dataset were performed as our previous study in Ramamoorthy et al. [8]. In the current study, the miRNA and mRNA expression profiles were obtained from primary human hepatocyte cultures (obtained from CellzDirect) from 7 donors, each treated with rifampin or vehicle for a total of 14 datasets. Cultures from each subject were treated as biologic replicates (*n* = 7). The hepatocytes were treated with rifampin or vehicle for 24 h and the total RNAs were isolated using a miRNeasy kit. The mRNA expression profile included 12,780 genes. The miRNA expression profile, which included 334 miRNAs, was measured using the Taqman OpenArray Human miRNA Panel using a NT Cycler. The mRNAs expression was measured using a standard method including EZBead preparation, Next-Gene sequencing, read quality assessment, sequence alignment, and RNA-Seq differential expression analysis.

### Construction of RrPIN

The PPI data was downloaded from the Human Protein Reference Database (HPRD) [13], which contains experimentally validated interactions within the human proteome. The human liver protein interaction network (HLPN) [14] contains proteome-scale protein interaction maps of the human liver. It is comprised of 3484 interactions among 2582 proteins and provides substantial new insights into systems biology, disease research, and drug discovery. To construct the human protein interaction network (HPIN), all the proteins and non-overlapped interactions in the HPLN and HPRD were merged as the nodes and interactions of HPIN.

To construct the rifampin-regulated gene network, we integrated the gene expression profile and HPIN as follows: the SDGs which were included in the HPIN were used as RrPIN’s nodes, and the interactions of RrPIN’s nodes in the HPIN were used as the RrPIN’s interactions. Cytoscape version 3.0.2 software (http://chianti.ucsd.edu/cytoscape-3.2.0/) [15] was used to generate the network.

### Identification of the functional modules

Particular interest of BioNet and jActiveModules were the identification of functional modules in the network in which the nodes have significant P-values by means of detecting differentially expressed regions in networks. This indicates a group of nodes which are densely connected and have significant differences in expression level, suggesting a module whose activity is influenced by the experimental context of the expression data. The functional modules tend to correspond to shared common cellular function beyond the scope of classical pathways [16–18]. The maximally scoring optimal module was identified using BioNet [17, 18]. And the jActiveModules plug-in of cytoscape was used to further identify multiple significant modules in the PPI network [16].

### Enrichment analysis of functional modules

The gene-annotation enrichment analysis was performed using the Database for Annotation, Visualization, and Integrated Discovery (DAVID), which provides a comprehensive set of functional annotation tools for biological interpretation of large gene lists. GO and KEGG are included in the set of functional annotation tools of DAVID. To study the rifampin-regulated biological process, we used DAVID’s GOTERM_BP_FAT (lower levels of biological process ontology), and KEGG pathway analysis to identify enriched biological themes, particularly GO terms [19].

### Identification of miRNAs and analysis of their functions

MiRNAs with *p* < 0.05 were regarded as significant differentially expressed miRNAs. We identified these miRNAs’ target genes using the R library RmiR.Hs.miRNA [20] which collects information from different miRNA target databases. In this study, Targetscan [21, 22], miRanda [23], PicTar [24] and miRTarBase [25, 26] were choosen. The BiomaRt [27] library, which provides a wide range of online queries from gene annotation to database mining, was used to convert gene IDs to gene symbols based on the hsapiens_gene_ensembl database. For each miRNA, the miRNA-targeted genes belonging to functional modules were considered as the nodes of the miRNA-regulated PIN. The interactions of these genes in PPI and of each miRNA with its target genes were considered as the interactions of the miRNA-regulated PIN. As a result, we obtained the miRNA-regulated PIN. For each miRNA, we analysed its potential functions by analysing the miRNA’s target genes based on GO and KEGG.

## Results

### SDGs and RrPIN

**Fig. 2:**
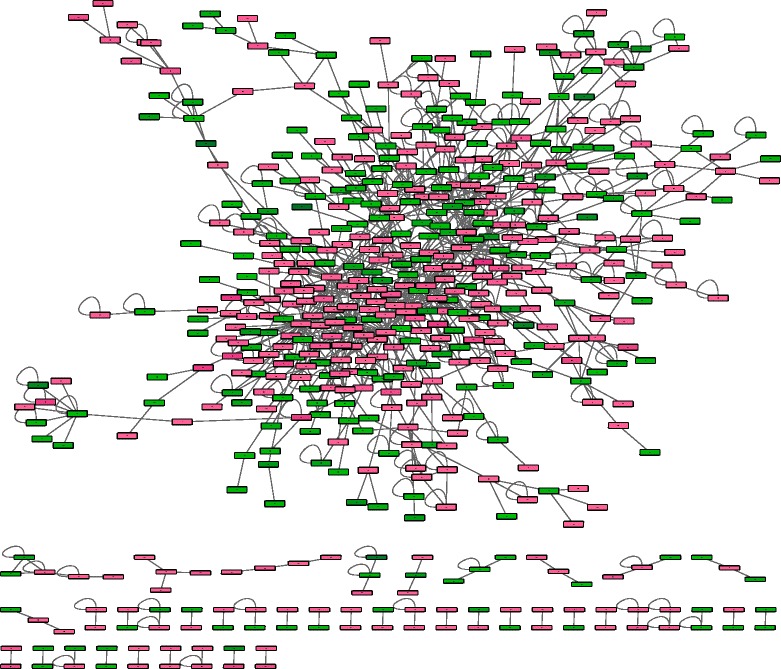
The workflow of the RrPIN. Color is according to the fold change where red denotes upregulated and green denotes downregulated

The mRNA expression profile was obtained from RNA-seq data from primary hepatocytes from 7 subjects treated with rifampin or vehicle. In order to focus on cellular responses that are triggered by the rifampin treatment, we pre-selected the genes that are differentially expressed with a loose *p*-value threshold at *p*-value < 0.01 without multiple hypothesis correction. Our further analysis focuses on 1866 differentially expressed genes that pass the threshold. We mapped all the differentially expressed genes on the combined human protein interaction network, which consists of 10,210 proteins with 42,521 interactions. As shown in Fig. 2, the resultant rifampin interaction network includes 663 proteins with 1024 interactions.

### Identification and analysis of the functional modules

The aforementioned network contains candidate differentially expressed genes with a flexible *p*-value cutoff. This is intentional since our network analysis will be further used to identify a cluster of interacting molecules that tend to be collectively differentially expressed, and therefore will reduce false positives. We used BioNet [17, 18], a bioconductor package for the functional analysis of biological networks, which uses the *p*-values obtained from differential expressed genes from RNA-seq data. The goal of this algorithm is to identify functional modules, or significantly differentially expressed subnetworks, within large networks [17]. This was achieved by computing a score for each node, reflected by its *p*-value, and used a network search algorithm to find the highest-scoring subgraph.

In this study, the maximally functional module was identified by computing optimal scores based on the *p*-values from the RNA-seq data to evaluate how molecular activity changes were correlated with rifampin regulation. False discovery rate (FDR) is an adjustment parameter for controlling the resultant subnetwork size. Since FDR can be used for fine-tuning of the signal noise decomposition, we scan a large range of FDRs and evaluate the obtained modules according to true-positive rate and precision (ratio of true positives to all positively classify). As a result, a threshold value of >0.0001 was used, because others thresholds lead to either too small or too large size of the module. The derived module captures the characteristically differently expressed interactions associated with rifampin treatment. There were 84 genes and 89 interactions in the maximally functional module. *P*-values, fold-changes, and false discovery rates (FDR) for the genes of the maximally functional module are shown in Additional file 1.

**Fig. 3:**
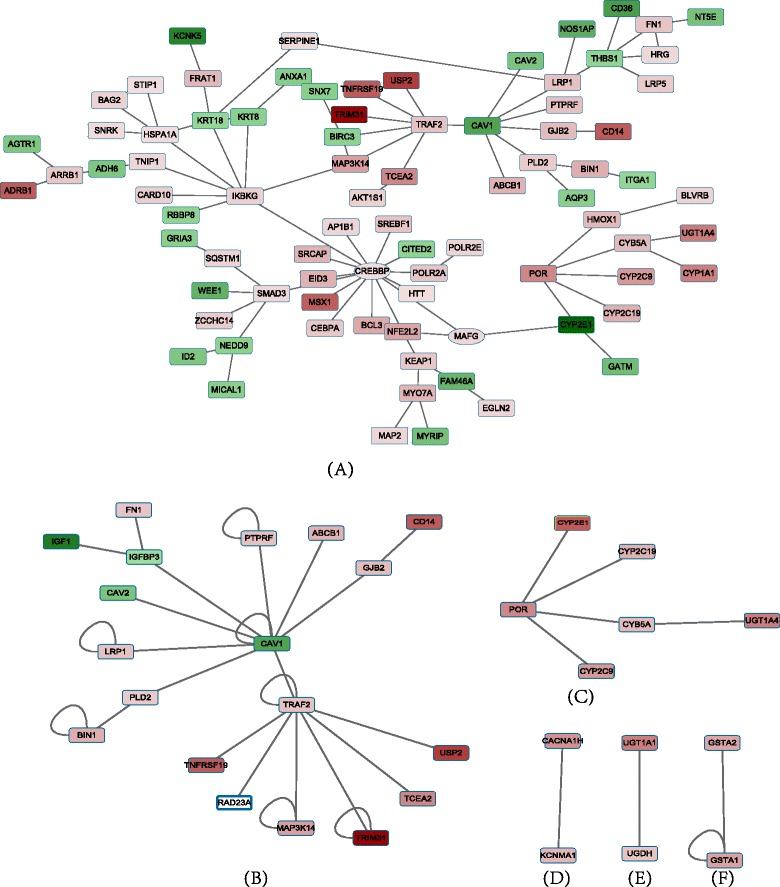
The functional modules of RrPIN. **a** The maximally functional module of PPI network. Color is according to the fold change where red denotes upregulated and green denotes downregulated. The shape of the nodes depicts the aggregate score: circles indicate a negative score, rectangles denotes a positive score. **b**, **c**, **d**, **e** and **f** are the five functional modules of RrPIN. The regulatory relationships are denoted by colours in which red indicates upregulated genes, and green indicates downregulated. As well, the depth of the colour explains the size of fold change

To avoid bias and to ensure generality of our results, besides the maximally functional module, we identified the multiple functional modules and key genes that also demonstrated the significant enrichment on differentially expressed genes; this analysis was done using the jActiveModules plug-in in cytoscape [15]. The maximum depth from the start node was set as 2 and the overlap threshold was set as 0. There were 31 nodes and 36 interactions within the five functional modules. *P*-values, Fold Changes, and false discovery rates (FDR) for the genes within the five functional modules are shown in Additional file 2. The maximally functional module and five functional modules are shown in Fig. 3.

As expected, the results from BioNet are essentially in agreement with the results from the jActiveModules plug-in. The maximally functional module included all the nodes and interactions of the five functional modules.

### Enrichment analysis of functional modules

To systematically determine the roles of genes in the functional modules, the online biological classification tool DAVID was used to carry out the functional classification based on GO and key signal pathways from KEGG. Since most of the genes within the five modules were included in the maximally functional module, we primarily focused on the analysis of the maximally functional module. Table of top 20 GO terms and top 10 KEGG terms for the genes of functional modules were shown in Table 1. Due to the redundant nature of the ontology analysis, functional annotation clustering was also derived from DAVID. A table of the top 20 functional annotations clustering for the genes of functional modules assessed by DAVID are provided in Additional file 3. Since the evidence suggests that rifampin have a broad spectrum of effect on enhancing drug metabolism, specifically, we focused on the GO terms “drug” and “metabolism,” and the top six listed KEGG pathways were extracted and the duplicates were eliminated. The *p*-value was used to evaluate the significance of the GO terms and KEGG pathways. Table 2 shows the results of the enrichment analysis of the maximally functional module in the RrPIN.

**Table 1:** Table of top 20 GO terms and top 10 KEGG terms for the genes of functional modules

Category	Term	Count	Percent	*P*-value	Benjiamini
GOTERM_BP_FAT	regulation of apoptosis	19	22.6	5.10E-07	7.30E-04
GOTERM_BP_FAT	regulation of programme cell death	19	22.6	5.90E-07	4.20E-04
GOTERM_BP_FAT	regulation of cell death	19	22.6	6.20E-07	3.00E-04
GOTERM_BP_FAT	negative regulation of apoptosis	13	15.5	7.90E-07	2.80E-04
GOTERM_BP_FAT	negative regulation of programmed cell death	13	15.5	9.10E-07	2.60E-04
GOTERM_BP_FAT	negative regulation of cell death	13	15.5	9.40E-07	2.20E-04
GOTERM_BP_FAT	membrane organization	13	15.5	1.70E-06	3.50E-04
GOTERM_BP_FAT	vesicle-mediated transport	15	17.9	4.50E-06	8.10E-04
GOTERM_BP_FAT	membrane invagination	10	11.9	4.70E-06	7.40E-04
GOTERM_BP_FAT	endocytosis	10	11.9	4.70E-06	7.40E-04
GOTERM_BP_FAT	response to hypoxia	8	9.5	1.20E-05	1.70E-03
GOTERM_BP_FAT	response to oxygen levels	8	9.5	1.60E-05	2.10E-03
GOTERM_BP_FAT	response to inorganic substance	9	10.7	2.30E-05	2.70E-03
GOTERM_BP_FAT	anti-apoptosis	9	10.7	2.40E-05	2.60E-03
GOTERM_BP_FAT	positive regulation of multicellular organismal process	9	10.7	7.90E-05	8.00E-03
GOTERM_BP_FAT	drug metabolic process	4	4.8	9.80E-05	9.20E-03
GOTERM_BP_FAT	response to metal ion	7	8.3	9.80E-05	8.70E-03
GOTERM_BP_FAT	phagocytosis	5	6	1.60E-04	1.40E-02
GOTERM_BP_FAT	response to organic substance	14	16.7	2.20E-04	1.80E-02
GOTERM_BP_FAT	regulation of tube size	5	6	2.40E-04	1.80E-02
KEGG_PATHWAY	Metabolism of xenobiotics by cytochrome P450	6	7.1	2.00E-04	1.70E-02
KEGG_PATHWAY	Retinol metabolism	5	6	1.40E-03	6.00E-02
KEGG_PATHWAY	Drug metabolism	5	6	2.40E-03	6.70E-02
KEGG_PATHWAY	Linoleic acid metabolism	3	3.6	2.70E-02	4.50E-01
KEGG_PATHWAY	Pathways in cancer	8	9.5	2.90E-02	4.00E-01
KEGG_PATHWAY	Focal adhesion	6	7.1	3.70E-02	4.10E-01
KEGG_PATHWAY	Porphyrin and chlorophyll metabolism	3	3.6	3.70E-02	3.70E-01
KEGG_PATHWAY	Small cell lung cancer	4	4.8	4.20E-02	3.70E-01
KEGG_PATHWAY	ECM-receptor interaction	4	4.8	4.20E-02	3.70E-01
KEGG_PATHWAY	TGF-beta signaling pathway	4	4.8	4.60E-02	3.60E-01

**Table 2:** Enrichment analysis of the maximally functional module in rifampin

DAVID (Term)	Genes	*P*-value
GO: Response to drug	ABCB1,UGT1A4,CAV1,CAV2	3.6E-2
KEGG: Metabolism of xenobiotics by cytochrome P450	UGT1A4,ADH6,CYP1A1,CYP2C19,CYP2C9,CYP2E1	2.0E-4
KEGG: Retinol metabolism	UGT1A4,ADH6,CYP1A1,CYP2C19,CYP2C9	1.4E-3
KEGG: Drug metabolism	UGT1A4,ADH6, CYP2C19,CYP2C9,CYP2E1	2.4E-3
KEGG: Linoleic acid metabolism	CYP2C19,CYP2C9,CYP2E1	2.7E-2
KEGG: Pathways in cancer	CEBPA,CREBBP,SMAD3,TRAF2,BIRC3,EGLN2,FN1,IKBKG	2.9E-2
Focal adhesion	BIRC3,CAV1,CAV2,FN1,ITGA1,THBS1	3.7E-2

The results show that the maximally functional module is relevant with seven functional enrichment terms: response to drug, metabolism of xenobiotics by cytochrome P450, retinol metabolism, drug metabolism, linoleic acid metabolism, cancer pathways, and focal adhesion. Among these terms, retinol metabolism, drug metabolism and linoleic acid metabolism contained many similarities in genes, since these three function terms were functionally correlated and clustered in functional annotation clustering in DAVID. In particular, the function pathways coincided with previously reported rifampin-induced biological functions. For example, rifampin affected the hepatic drug disposition and metabolism [28, 29] and it was a potent inducer of drug-metabolizing enzymes [6, 29–31]. Rifampin is also an inhibitor which rapidly downregulates angiogenesis and mitogenesis-related genes to target cancer cells [12, 32, 33].

In addition, we define the percentage of identified SDGs in each pathway relative to the total number of SDGs as pathway relative abundance. Assume that there are N SDGs-associated biological pathways, for i–th pathway, S(i) is number of identified SDGs, and N(i) is enriched number of the total SDGs. The pathway relative abundance E(x) is defined as:

1 $$ \mathrm{E}\left(\mathrm{i}\right)=\frac{\mathrm{S}\left(\mathrm{i}\right)}{\mathrm{N}\left(\mathrm{i}\right)}\kern0.24em \mathrm{i}\in 1..\mathrm{N} $$

**Fig. 4:**
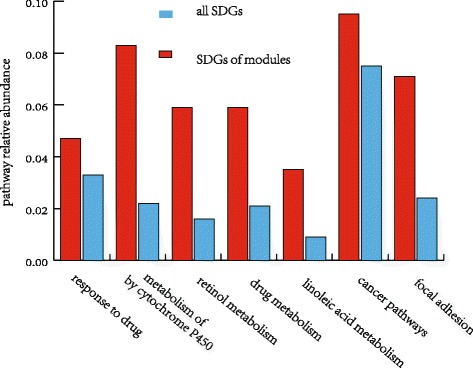
The pathway relative abundance of maximally functional module’s genes and all SDGs on the associated seven functional enrichment terms

Figure 4 shows the pathway relative abundance of maximally functional module’s genes and all SDGs on the associated seven functional enrichment terms. It can be seen that the SDGs of maximally functional module are more enriched and representative on each terms than the total SDGs. This suggests that our strategy in integrating PPI network with the differential expression analysis helped us in capturing more biologically relevant signals.

**Fig. 5:**
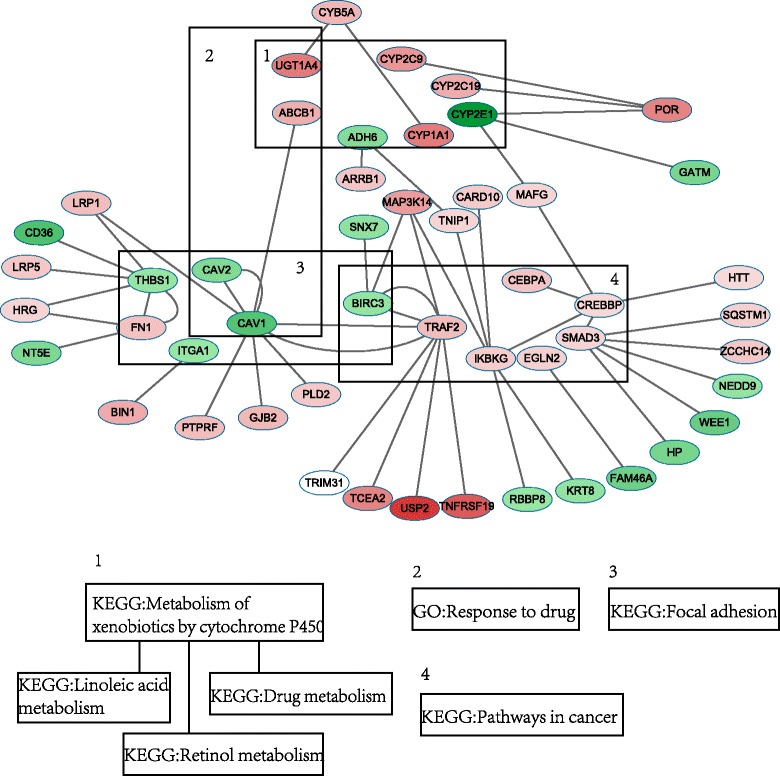
The RrPIN extension network of 19 genes and associated 7 functions

To analyze each functional enrichment term, we focus on the analysis of their genes. There were 19 key genes associated with 7 functions that we extracted using DAVID. Then we extend the protein interaction network of 19 genes based on the RrPIN with one level interaction. The RrPIN extension network of 19 genes consists of 50 nodes and 53 interactions. The RrPIN extension network of 19 genes and associated 7 functions are shown as Fig. 5.

It is worth noting that *UGT1A4, ADH6, CYP1A1, CYP2C19, CYP2C9* and *CYP2E1* are all associated with metabolism of xenobiotics, drug metabolism, retinol metabolism, and linoleic acid metabolism. *BIRC3, CAV1, CAV2, FN1, ITGA1* and *THBS1* were functionally enriched to focal adhesion, which contributes to antiangiogenic and anti-tumour effects. These results indicate that rifampin induced drug metabolism, partially, by regulating *UGT1A4, ADH6, CYP1A1, CYP2C19, CYP2C9* and *CYP2E1*. These results also signify that rifampin can influence the anti-angiogenesis and anti-tumour effects of drugs by regulating *BIRC3, CAV1, CAV2, FN1, ITGA1* and *THBS1*. Previous reports support these findings, stating that *UGT1A4 CYP1A1, CYP2C19, CYP2C9* and *CYP2E1* are drug-metabolizing enzymes [34, 35], and *ADH6* modulates the risk for drug dependence [35]. *BIRC3* contains anti-apoptotic genes, which can be suppressed to counteract cancerous activity [36]. *CAV1* and *CAV2* were correlated with tumour growth and metastasis [37–39], and *FN1* was a potential biomarker for some cancers [40, 41], while *ITGA1* and *THBS1* were also associated with cancer risk [42, 43].

In addition, some of the 19 key genes were hub proteins that interacted with multiple proteins. For example, *CAV1, CREBBP, SMAD3, TRAF2, KBKG* and *THBS1* had at least four interactions with other proteins. These results suggest that these six genes are important components in biological pathways regulated by rifampin.

### Joint analysis of key genes and associated miRNAs

To identify miRNAs that may regulate these key genes within the functional modules, we correlated the alterations in the miRNA and gene expression. In this process, we extracted the significant differentially expressed miRNAs (*p* < 0.05), and identified 20 miRNAs. The significant differentially expressed miRNAs are shown in Table 3.

**Table 3:** The significant differentially expressed miRNAs

miRNA	*P*-value	miRNA	*P*-value
Upregulated		Upregulated	
miR-886-3p	0.0002	miR-660	0.0297
miR-766	0.0075	miR-638	0.0302
miR-92a	0.0169	miR-25	0.0338
miR-107	0.0177	miR-616	0.0446
miR-30d#	0.0195	miR-576-3p	0.0453
miR-335	0.0241	miR-218	0.0499
Downregulated		Downregulated	
miR-186	0.0018	miR-320	0.0376
miR-361	0.0111	miR-202	0.0396
miR-95	0.0219	miR-200b#	0.0426
miR-345	0.0239	let-7 g	0.0435

**Fig. 6:**
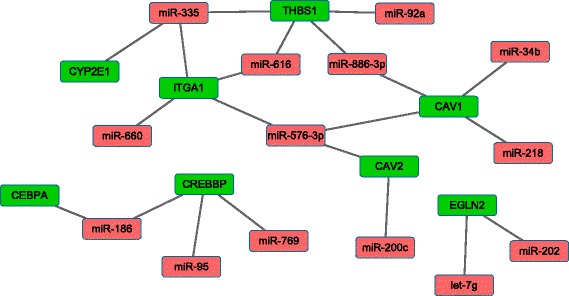
The miRNA-regulated PIN which is constructed by the genes of functional modules

In order to identify the target genes of significant differentially expressed miRNAs, three databases (Targetscan, miRanda and PicTar) were used [20–23]. The miRNA-mRNA pairs were extracted for each significant differentially expressed miRNA. In order to include verified miRNA-mRNA pairs, we also extracted the miRNA-mRNA pairs from the miRTarBase [25, 26]. And the miRNA-mRNA pairs of which mRNA is a gene of maximally functional module were chosen. Then, we established the miRNA-regulated PIN, which showed a negative correlation between the miRNA and the mRNA. The miRNA-regulated PIN, which is constructed of the genes in the functional modules, is shown in Table 4 and Fig. 6.

**Table 4:** The miRNA-regulated PIN which constructed by the genes of functional modules

Gene list	logFC	miRNA	*P*-value	Fold change
CYP2E1	−1.4341	miR-335	0.0242	1.3300
CAV1	−0.8518	miR-34b	0.1753	185.3764
		miR-886-3p	0.0001	1.5645
		miR-218	0.0499	1.9012
		miR-576-3p	0.0453	2.1916
CAV2	−0.5386	miR-200c	0.0913	4.8313
		miR-576-3p	0.0453	2.1916
CEBPA	0.5812	miR-186	0.0017	0.8356
CREBBP	0.3821	miR-186	0.0017	0.8356
		miR-95	0.0216	0.6320
		miR-769	0.1249	0.8388
EGLN2	0.4574	miR-202	0.0396	0.5988
		let-7 g	0.0435	0.8402
ITGA1	−0.3754	miR-616	0.0446	1.3337
		miR-660	0.0297	1.2642
		miR-576-3p	0.0453	2.1916
		miR-335	0.0242	1.3300
THBS1	−0.3951	miR-886-3p	0.0001	1.5645
		miR-335	0.0242	1.3300
		miR-616	0.0446	1.3337
		miR-92a	0.0169	1.1319

Eight genes and 14 miRNAs were identified to have significant differential expression changes. Each of these genes was regulated by multiple miRNAs. Due to the miRNAs control of gene expression, either by degradation of the target mRNAs or by inhibition of protein translation, miRNA-regulated PPI networks can uncover new rules of miRNA regulation or protein interaction. Thus, we predicted the potential functions of miRNAs based on the function of their target genes as shown in Table 5.

**Table 5:** The potential functions of miRNAs

DAVID (Term)	miRNA
GO: Response to drug	miR-34b, miR-886-3p, miR-218, miR-576-3p, miR-200c
KEGG: Metabolism of xenobiotics by cytochrome P450	miR-335
KEGG: Drug metabolism	miR-335
KEGG: Linoleic acid metabolism	miR-335
KEGG: Pathways in cancer	miR-186, miR-95, miR-769
Focal adhesion	miR-34b, miR-886-3p, miR-218, miR-576-3p, miR-200c, miR-616, miR-660, miR-335, miR-92a

Twelve miRNAs were extracted which associated with 6 biological pathways including response to drug, metabolism of xenobiotics by cytochrome P450, drug metabolism, linoleic acid metabolism, cancer pathways, and focal adhesion through regulation of 8 target genes. The results suggest that miR-335 influences drug metabolism through negative regulation of *CYP2E1*, which is a drug metabolizing enzyme that is affected by rifampin treatment. Therefore, it is possible that rifampin may alter miRNA expression, which in turn affects the expression of the drug metabolizing enzyme gene *CYP2E1*. MiR-186 was found to regulate two genes (*CEBPA, CREBBP*), which were associated with cancer pathways. MiR-186, miR-769, miR-95, miR-202 and let-7 g were also relevant to cancer pathways, but did not serve other functions. Previous studies have demonstrated that rifampin also inhibited anti-angiogenesis by regulating the expression of multiple miRNAs (miR-34b, miR-886-3p, miR-218, miR-576-3p, miR-200c, miR-616, miR-660, miR-335, miR-92a), and further induced the gene expression of *BIRC3, CAV1, CAV2, FN1, ITGA1* and *THBS1*.

## Conclusions

In conclusion, a novel integrative network-based method was used to identify the functional modules and discover the potential functions of miRNAs based on human protein network, mRNA and miRNA expression profile in rifampin treated hepatocytes. Furthermore, this method identifies 19 genes and 7 crucial biological pathways. By analysing the miRNA-regulated PIN, we suggested that 12 miRNAs were associated with 6 biological pathways through regulation of 8 target genes. Our results suggest that rifampin contributes to changes in the expression of genes and miRNAs, and induces multiple biological pathways. This study not only provides an insight into functional modules that are associated with rifampin-treated human hepatocytes in human protein interaction network, it also shows that the integrated analysis of mRNA, miRNA expression profile and PIN can be used to study the molecular mechanism of rifampin-induced drug disposition.

## Additional files

Additional file 1:
*P*-values, Fold Change and false discovery rates (FDR) for the genes of the maximally functional module. (PDF 161 kb) Additional file 2:
*P*-values, Fold Change and false discovery rates (FDR) for the genes of the five functional modules. (PDF 105 kb) Additional file 3: Top 10 GO terms and KEGG terms for the genes in functional modules from the DAVID were provided as Additional file 3. (PDF 264 kb)
