# Supplementary material for: Identification of rifampin-regulated functional modules and related microRNAs in human hepatocytes based on the protein interaction network
Source: BMC Genomics. 2016 Aug 22;17(Suppl 7):517. doi: 10.1186/s12864-016-2909-6 (PMC5001204; doi:10.1186/s12864-016-2909-6)
Supplement: Additional file 1: — P-values, Fold Change and false discovery rates (FDR) for the genes of the maximally functional module. (PDF 161 kb) [file 12864_2016_2909_MOESM1_ESM.pdf]

Row.names	logFC	logCPM	LR	PValue	FDR	score
ABCB1	0.781842	8.247882	74.32397	6.63E-18	9.01E-16	29.31959
ADH6	-0.51482	7.192559	30.93753	2.66E-08	1.15E-06	8.637564
ADRB1	1.627262	1.193321	41.22681	1.36E-10	8.09E-09	13.57225
AGTR1	-0.5116	4.440014	21.18387	4.17E-06	0.000105	3.909815
AKT1S1	0.487082	3.376339	14.0089	0.000182	0.002771	0.378282
ANXA1	-0.45524	3.892246	13.80406	0.000203	0.003036	0.276354
AP1B1	0.444063	5.456307	19.60818	9.51E-06	0.000215	3.138723
AQP3	-0.42789	5.351138	17.61059	2.71E-05	0.000541	2.15928
ARRB1	0.577594	3.07078	16.40234	5.12E-05	0.000942	1.564233
BAG2	0.471139	5.358268	21.82267	2.99E-06	8.03E-05	4.220938
BCL3	0.864843	1.482152	18.00232	2.21E-05	0.000451	2.350041
BIN1	0.822391	4.289316	52.66569	3.95E-13	3.30E-11	19.03586
BIRC3	-0.41185	8.811137	20.94068	4.74E-06	0.000117	3.789983
BLVRB	0.464232	3.861911	14.17867	0.000166	0.002569	0.462701
CARD10	0.516718	3.839282	18.35077	1.84E-05	0.000382	2.521415
CAV1	-0.8518	6.351125	78.42201	8.32E-19	1.21E-16	31.26084
CAV2	-0.5386	7.080655	33.18784	8.37E-09	3.87E-07	9.719018
CD14	1.630916	5.953018	262.1085	5.96E-59	2.93E-56	117.7177
CD36	-0.89744	0.990976	13.77571	0.000206	0.003064	0.26224
CEBPA	0.581282	5.119956	30.98112	2.61E-08	1.13E-06	8.655312
CITED2	-0.39253	6.917221	17.68095	2.61E-05	0.000525	2.194446
CREBBP	0.382177	4.059073	10.85691	0.000984	0.010843	-1.20069
CYB5A	0.707906	9.318423	62.36751	2.85E-15	2.94E-13	23.64839
CYP1A1	1.26928	5.792901	119.8092	6.96E-28	1.44E-25	50.81038
CYP2C19	0.794506	7.990218	72.28439	1.86E-17	2.45E-15	28.35477
CYP2C9	1.012758	10.69292	126.41	2.50E-29	5.61E-27	53.92165
CYP2E1	-1.43406	7.783496	232.2929	1.89E-52	8.03E-50	103.7166
EGLN2	0.4574	3.718154	13.859	0.000197	0.002969	0.303699
EID3	0.78844	3.13823	32.47515	1.21E-08	5.51E-07	9.37431
FAM46A	-0.67256	6.570705	50.25176	1.35E-12	1.06E-10	17.8864
FN1	0.605011	11.15734	46.17581	1.08E-11	7.35E-10	15.94148
FRAT1	0.735218	2.843312	25.01537	5.69E-07	1.80E-05	5.77275
GATM	-0.59971	11.3772	45.56342	1.48E-11	9.89E-10	15.64678
GJB2	0.692627	5.829275	49.8689	1.64E-12	1.27E-10	17.70439
GRIA3	-0.43008	4.360965	14.42511	0.000146	0.002302	0.58515
HMOX1	0.712809	3.455651	30.70411	3.01E-08	1.28E-06	8.521947
HP	-0.60141	11.68125	45.67201	1.40E-11	9.45E-10	15.69876
HRG	0.418161	6.456386	19.27369	1.13E-05	0.00025	2.977421
HSPA1A	0.382442	5.314084	13.81043	0.000202	0.003033	0.279522
HTT	0.373734	6.073422	15.04474	0.000105	0.001738	0.892547
ID2	-0.51691	6.925046	30.67253	3.05E-08	1.30E-06	8.509599
IKBKG	0.491087	3.287642	13.80327	0.000203	0.003036	0.275963
ITGA1	-0.37537	7.174065	16.46376	4.96E-05	0.000917	1.593927

KCNK5	-1.13739	3.660467	70.14849	5.50E-17	6.82E-15	27.34074
KEAP1	0.582621	4.156287	25.81962	3.75E-07	1.24E-05	6.16273
KRT18	-0.36685	7.972157	16.28497	5.45E-05	0.000998	1.505812
KRT8	-0.42013	8.594638	21.52194	3.50E-06	9.07E-05	4.073637
LRP1	0.635707	5.39105	37.88987	7.49E-10	3.99E-08	11.97654
LRP5	0.543532	3.646617	18.94682	1.34E-05	0.000287	2.817996
MAFG	0.426676	3.972673	13.04065	0.000305	0.004215	-0.10428
MAP2	0.448175	4.15576	13.53696	0.000234	0.003404	0.143314
MAP3K14	0.963198	2.708224	40.36074	2.11E-10	1.22E-08	13.16146
MICAL1	-0.42479	4.247371	13.66213	0.000219	0.00322	0.205678
MSX1	1.58731	0.224097	26.33631	2.87E-07	9.80E-06	6.412872
MYO7A	0.750208	4.603307	46.98494	7.15E-12	4.91E-10	16.32723
MYRIP	-0.57456	4.278972	25.06049	5.56E-07	1.77E-05	5.794367
NEDD9	-0.40566	8.096531	19.95967	7.91E-06	0.000183	3.311021
NFE2L2	0.90388	7.218413	94.16677	2.90E-22	4.94E-20	38.70748
NOS1AP	-0.74828	3.203347	29.54472	5.46E-08	2.17E-06	7.964964
NT5E	-0.55848	5.901211	32.55995	1.16E-08	5.31E-07	9.41378
PLD2	0.549999	2.889249	14.46841	0.000143	0.002263	0.606654
POLR2A	0.611897	3.835053	25.20689	5.15E-07	1.67E-05	5.866012
POLR2E	0.469134	4.568353	18.64733	1.57E-05	0.000333	2.669838
POR	1.197264	6.768948	156.9173	5.34E-36	1.42E-33	68.28717
PTPRF	0.639366	6.436931	45.21186	1.77E-11	1.17E-09	15.47942
RBBP8	-0.38942	5.775735	15.86234	6.81E-05	0.001207	1.297448
SERPINE1	0.399277	9.802528	19.97795	7.83E-06	0.000182	3.320529
SMAD3	0.42998	4.504394	15.20517	9.64E-05	0.001624	0.972402
SNRK	0.387346	5.996178	16.13447	5.90E-05	0.001063	1.431608
SNX7	-0.43596	5.40253	18.67752	1.55E-05	0.000329	2.681829
SQSTM1	0.464945	9.119435	26.97471	2.06E-07	7.34E-06	6.723025
SRCAP	0.771987	3.559544	36.27857	1.71E-09	8.74E-08	11.20443
SREBF1	0.619251	4.518346	31.41034	2.09E-08	9.17E-07	8.863124
STIP1	0.363492	7.059521	15.38659	8.76E-05	0.001501	1.061934
TCEA2	1.194078	2.840003	57.36776	3.61E-14	3.30E-12	21.27367
THBS1	-0.39505	6.26565	15.97468	6.42E-05	0.001146	1.352607
TNFRSF19	1.605388	3.345264	102.7133	3.87E-24	7.17E-22	42.74484
TNIP1	0.426098	6.621468	20.65746	5.49E-06	0.000132	3.652595
TRAF2	0.633813	3.104377	21.41362	3.70E-06	9.50E-05	4.021662
TRIM31	2.498618	1.829416	120.8331	4.16E-28	8.85E-26	51.29175
UGT1A4	1.295465	10.19577	202.9222	4.81E-46	1.81E-43	89.92115
USP2	1.924259	0.738857	49.79502	1.71E-12	1.31E-10	17.6653
WEE1	-0.71408	6.7836	57.95013	2.69E-14	2.51E-12	21.54881
ZCCHC14	0.544027	4.22095	22.55632	2.04E-06	5.69E-05	4.578528
