# Supplementary material for: Identification of rifampin-regulated functional modules and related microRNAs in human hepatocytes based on the protein interaction network
Source: BMC Genomics. 2016 Aug 22;17(Suppl 7):517. doi: 10.1186/s12864-016-2909-6 (PMC5001204; doi:10.1186/s12864-016-2909-6)
Supplement: Additional file 2: — P-values, Fold Change and false discovery rates (FDR) for the genes of the five functional modules. (PDF 105 kb) [file 12864_2016_2909_MOESM2_ESM.pdf]

moudule	name	logCPM	logFC	LR	pval	FDR
1	RAD23A	3.909317	0.336953	7.8921	0.004965	0.236908
1	BIN1	4.289316	0.822391	52.66569	3.95E-13	3.30E-11
1	IGF1	5.695979	-1.1966	139.6325	3.20E-32	0.558752
1	CD14	5.953018	1.630916	262.1085	5.96E-59	2.93E-56
1	FN1	11.15734	0.605011	46.17581	1.08E-11	1.87E-06
1	PLD2	2.889249	0.549999	14.46841	1.43E-04	0.615714
1	LRP1	5.39105	0.635707	37.88987	7.49E-10	0.304046
1	CAV2	7.080655	-0.5386	33.18784	8.37E-09	3.87E-07
1	PTPRF	6.436931	0.639366	45.21186	1.77E-11	0.05891
1	ABCB1	8.247882	0.781842	74.32397	6.63E-18	9.01E-16
1	TNFRSF19	3.345264	1.605388	102.7133	3.87E-24	0.689091
1	CAV1	6.351125	-0.8518	78.42201	8.32E-19	1.21E-16
1	TRIM31	1.829416	2.498618	120.8331	4.16E-28	0.277165
1	TCEA2	2.840003	1.194078	57.36776	3.61E-14	0.270409
1	TRAF2	3.104377	0.633813	21.41362	3.70E-06	0.607507
1	GJB2	5.829275	0.692627	49.8689	1.64E-12	0.752952
1	USP2	0.738857	1.924259	49.79502	1.71E-12	0.867063
1	MAP3K14	2.708224	0.963198	40.36074	2.11E-10	0.185909
2	CYP1A1	5.792901	1.26928	119.8092	6.96E-28	1.44E-25
2	POR	6.768948	1.197264	156.9173	5.34E-36	0.533644
2	CYP2E1	7.783496	-1.43406	232.2929	1.89E-52	8.03E-50
2	CYP2C9	10.69292	1.012758	126.41	2.50E-29	5.61E-27
2	CYB5A	9.318423	0.707906	62.36751	2.85E-15	2.94E-13
2	CYP2C19	7.990218	0.794506	72.28439	1.86E-17	2.45E-15
2	UGT1A4	10.19577	1.295465	202.9222	4.81E-46	2.04E-37
3	CACNA1H	3.288568	1.093152	61.55417	4.31E-15	4.30E-13
3	KCNMA1	4.570233	0.738706	39.82824	2.77E-10	1.54E-08
4	UGT1A1	10.41924	1.286226	201.2782	1.10E-45	4.01E-43
4	UGDH	9.411177	0.617806	47.35648	5.92E-12	4.16E-10
5	GSTA2	5.00096	0.900545	57.35624	3.64E-14	0.887722
5	GSTA1	6.315528	0.940992	91.09722	1.37E-21	0.261818
