# Supplementary material for: Identification of rifampin-regulated functional modules and related microRNAs in human hepatocytes based on the protein interaction network
Source: BMC Genomics. 2016 Aug 22;17(Suppl 7):517. doi: 10.1186/s12864-016-2909-6 (PMC5001204; doi:10.1186/s12864-016-2909-6)
Supplement: Additional file 3: — Top 10 GO terms and KEGG terms for the genes in functional modules from the DAVID were provided as Additional file 3. (PDF 264 kb) [file 12864_2016_2909_MOESM3_ESM.pdf]

Annotation Cluster 1	Enrichment Score:5.12	term	Count	P_Value	Benjamini
	GOTERM_CC_FAT	membrane fraction	20	1.80E-08	4.00E-06
	GOTERM_CC_FAT	insoluble fraction	20	3.30E-08	3.60E-06
	GOTERM_CC_FAT	cell fraction	22	7.50E-08	5.50E-06
	GOTERM_CC_FAT	microsome	11	3.90E-07	2.10E-05
	GOTERM_CC_FAT	vesicular fraction	11	5.10E-07	2.20E-05
	SP_PIR_KEYWORDS	transmembrane protein	14	3.50E-06	2.50E-04
	SP_PIR_KEYWORDS	membrane	30	3.60E-01	8.40E-01
	GOTERM_CC_FAT	intrinsic membrane	21	9.90E-01	1.00E+00
Annotation Cluster 2	Enrichment Score:4.76				
	GOTERM_BP_FAT	membrane organization	13	1.70E-06	3.50E-04
	GOTERM_BP_FAT	vesicle-mediated transport	15	4.50E-06	8.10E-04
	GOTERM_CC_FAT	cytoplasmic vesicle	15	4.70E-06	1.70E-04
	GOTERM_BP_FAT	endocytosis	10	4.70E-06	7.40E-04
	GOTERM_BP_FAT	membrane invagination	10	4.70E-06	7.40E-04
	GOTERM_CC_FAT	vesicle	15	7.60E-06	2.40E-04
	GOTERM_BP_FAT	phagocytosis	5	1.60E-04	1.40E-02
	SP_PIR_KEYWORDS	Endocytosis	4	8.30E-03	7.30E-02
Annotation Cluster 3	Enrichment Score:4.14				
	GOTERM_CC_FAT	cytoplasmic vesicle	15	4.70E-06	1.70E-04
	GOTERM_CC_FAT	vesicle	15	7.60E-06	2.40E-04
	GOTERM_CC_FAT	cytoplasmic membrane-bounded vesicle	12	1.20E-04	3.30E-03
	GOTERM_CC_FAT	membrane-bounded vesicle	12	1.60E-04	3.90E-03
	GOTERM_CC_FAT	cytoplasmic vesicle part	6	2.90E-03	3.20E-02
Annotation Cluster 4	Enrichment Score:3.95				
	GOTERM_BP_FAT	regulation of apoptosis	19	5.10E-07	7.30E-04
	GOTERM_BP_FAT	regulation of programmed cell death	19	5.90E-07	4.20E-04
	GOTERM_BP_FAT	regulation of cell death	19	6.20E-07	3.00E-04

	GOTERM_BP_FAT	negative regulation apoptosis	13	7.90E-07	2.80E-04
	GOTERM_BP_FAT	negative regulation cell death	13	9.10E-07	2.60E-04
	GOTERM_BP_FAT	negative regulation cell death	13	9.40E-07	2.20E-04
	GOTERM_BP_FAT	anti-apoptosis	9	2.40E-05	2.60E-03
	GOTERM_BP_FAT	positive regulation of apoptosis	10	8.00E-04	4.60E-02
	GOTERM_BP_FAT	positive regulation of programmed cell death	10	8.40E-04	4.70E-02
	GOTERM_BP_FAT	positive regulation of programmed cell death	10	8.60E-04	4.60E-02
	GOTERM_BP_FAT	protein kinase	9	1.30E-03	5.70E-02
	GOTERM_BP_FAT	induction of cascade apoptosis	7	1.00E-02	2.00E-01
	GOTERM_BP_FAT	induction of cascade cell death	7	1.00E-02	2.00E-01
	GOTERM_BP_FAT	regulation of cascade	4	5.80E-02	4.90E-01
	GOTERM_BP_FAT	regulation of cascade localization	3	4.20E-01	9.60E-01
Annotation Cluster 5	Enrichment Score: 2.96				
	GOTERM_CC_FAT	microsome	11	3.90E-07	2.10E-05
	GOTERM_CC_FAT	vesicular fraction	11	5.10E-07	2.20E-05
	SP_PIR_KEYWORDS	electron transfer	6	2.40E-06	2.60E-04
	SP_PIR_KEYWORDS	microsome	7	6.50E-06	2.80E-04
	SP_PIR_KEYWORDS	metalloprotein	7	1.60E-05	5.80E-04
	UP_SEQ_FEATURE	metal ion-binding site:Iron (heme axial ligand)	6	6.80E-05	3.90E-02
	GOTERM_MF_FAT	oxygen binding	5	9.60E-05	2.90E-02
	GOTERM_BP_FAT	drug metabolic process	4	9.80E-05	9.20E-03
	SP_PIR_KEYWORDS	chromoprotein	5	1.50E-04	3.60E-03
	SP_PIR_KEYWORDS	heme	6	1.80E-04	3.90E-03
	KEGG_PATHWAY	Metabolism of xenobiotics by cytochrome P450	6	2.00E-04	1.70E-02
	GOTERM_MF_FAT	iron ion binding	9	3.20E-04	3.20E-02
	GOTERM_MF_FAT	heme binding	6	5.90E-04	4.40E-02
	PIR_SUPERFAMILY	PIRSF000045:cytoch	4	7.60E-04	5.10E-02

		rome P450 CYP2D6			
	GOTERM_MF_FAT	tetrapyrrole binding	6	7.80E-04	4.70E-02
	KEGG_PATHWAY	Retinol metabolism	5	1.40E-03	6.00E-02
	SP_PIR_KEYWORDS	iron	7	1.50E-03	2.60E-02
	INTERPRO	Cytochrome P450,E-class,group I	4	1.70E-03	3.70E-01
	KEGG_PATHWAY	Drug metabolism	5	2.40E-03	6.70E-02
	INTERPRO	Cytochrome P450,E-class,group I	4	2.50E-03	2.80E-01
	SP_PIR_KEYWORDS	oxidoreductase	9	2.90E-03	4.20E-02
	INTERPRO	Cytochrome P450	4	3.10E-03	2.50E-01
	INTERPRO	Cytochrome P450,conserved site	4	3.30E-03	2.00E-01
	GOTERM_BP_FAT	oxidation reduction	11	3.60E-03	1.00E-01
	SP_PIR_KEYWORDS	nadp	5	4.70E-03	5.50E-02
	SP_PIR_KEYWORDS	Monooxygenase	4	4.70E-03	5.30E-02
	COG_ONTOLOGY	Secondary metabolites biosynthesis,transport ,and catabolism	4	8.00E-03	7.70E-02
	GOTERM_MF_FAT	electron carrier activity	6	8.00E-03	2.60E-01
	GOTERM_CC_FAT	endoplasmic reticulum	12	1.10E-02	8.70E-02
	SP_PIR_KEYWORDS	endoplasmic reticulum	9	1.20E-02	9.80E-02
	GOTERM_MF_FAT	oxidoreductase activity,acting on paired donors,with incorporation reduction of molecular oxygen redulced flavin	3	1.20E-02	3.10E-01
	GOTERM_CC_FAT	extrinsic to membrane	8	1.50E-02	1.10E-01
	KEGG_PATHWAY	Linoleic acid metabolism	3	2.70E-02	4.50E-01
	KEGG_PATHWAY	Arachidonic acid metabolism	3	9.40E-02	5.70E-01
	GOTERM_CC_FAT	endoplasmic reticulum membrane	4	1.70E-01	5.50E-01
	GOTERM_CC_FAT	nuclear envelope-endoplasmi c reticulum network	4	1.90E-01	5.70E-01

	GOTERM_CC_FAT	endoplasmic reticulum part	4	2.70E-01	6.80E-01
Annotation Cluster 6	Enrichment Score:2.76				
	GOTERM_BP_FAT	regulation of response to external stimulus	7	3.10E-04	2.20E-02
	GOTERM_BP_FAT	regulation of blood coagulation	4	1.10E-03	5.50E-02
	GOTERM_BP_FAT	regulation of coagulation	4	1.70E-03	7.00E-02
	GOTERM_BP_FAT	negative regulation of multicellular organismal process	5	1.50E-02	2.30E-01
Annotation Cluster 7	Enrichment Score:2.72				
	GOTERM_BP_FAT	phagocytosis	5	1.60E-04	1.40E-02
	GOTERM_BP_FAT	apoptotic cell clearance	3	4.80E-04	3.10E-02
	GOTERM_CC_FAT	cell surface	7	9.60E-03	8.20E-02
	GOTERM_MF_FAT	lipoprotein binding	3	1.60E-02	3.70E-01
Annotation Cluster 8	Enrichment Score:2.42				
	SP_PIR_KEYWORDS	microsome	7	6.50E-06	2.80E-04
	GOTERM_BP_FAT	response to inorganic substance	9	2.30E-05	2.70E-03
	GOTERM_BP_FAT	response to metal ion	7	9.80E-05	8.70E-03
	GOTERM_BP_FAT	response to nutrient levels	7	9.50E-04	4.70E-02
	GOTERM_BP_FAT	response to nutrient	6	1.30E-03	5.90E-02
	GOTERM_BP_FAT	response to extracellular stimulus	7	1.70E-03	7.20E-02
	GOTERM_BP_FAT	response to calciumion	4	3.90E-03	1.10E-01
	GOTERM_BP_FAT	heterocycle catabolic process	4	9.90E-03	1.90E-01
	GOTERM_BP_FAT	porphyrin metabolic process	3	1.20E-02	2.10E-01
	GOTERM_BP_FAT	tetrapyrrole metabolic process	3	1.20E-02	2.10E-01
	GOTERM_BP_FAT	response to starvation	3	2.40E-02	3.10E-01
	GOTERM_BP_FAT	cellular response to nutrient levels	3	2.70E-02	4.50E-01
	GOTERM_BP_FAT	cellular response to	3	5.30E-02	3.20E-01

		extracellular stimulus			
	GOTERM_BP_FAT	cofactor metabolic process	3	3.10E-01	9.00E-01
	GOTERM_BP_FAT	steroid metabolic process	3	3.30E-01	9.10E-01
Annotation Cluster 9	Enrichment Score:2.3				
	GOTERM_BP_FAT	embryonic development ending in birth or egg hatching	8	3.10E-03	1.00E-01
	GOTERM_BP_FAT	in tuero embryonic development	6	3.50E-03	1.10E-01
	GOTERM_BP_FAT	chordate embrynic development	7	1.20E-02	2.10E-01
Annotation Cluster 10	Enrichment Score:2.29				
	GOTERM_BP_FAT	apoptosis	11	2.30E-03	9.00E-02
	GOTERM_BP_FAT	programmed cell death	11	2.60E-03	9.50E-02
	GOTERM_BP_FAT	cell death	12	2.70E-03	9.50E-02
	GOTERM_BP_FAT	death	12	2.80E-03	9.70E-02
	SP_PIR_KEYWORDS	Apoptosis	5	8.30E-02	4.00E-01
Annotation Cluster 11	Enrichment Score:2.12				
	GOTERM_CC_FAT	endomembrane system	13	6.70E-04	1.20E-02
	GOTERM_CC_FAT	organelle membrane	15	1.40E-03	2.00E-02
	GOTERM_CC_FAT	Golgi membrane	6	2.90E-03	3.30E-02
	GOTERM_CC_FAT	Golgi apparatus	11	1.50E-02	1.00E-01
	GOTERM_CC_FAT	Golgi apparatus part	6	1.90E-02	1.20E-01
	SP_PIR_KEYWORDS	golgi apparatus	5	2.50E-01	7.60E-01
Annotation Cluster 12	Enrichment Score: 2.04				
	GOTERM_CC_FAT	basolateral plasma membrane	7	6.60E-04	1.30E-02
	GOTERM_CC_FAT	focal adhesion	5	2.00E-03	2.70E-02
	GOTERM_CC_FAT	cell-substrate adherens junction	5	2.30E-03	2.90E-02
	GOTERM_CC_FAT	cell-substrate junction	5	2.80E-03	3.40E-02
	GOTERM_CC_FAT	adherens junction	5	8.80E-03	7.80E-02
	GOTERM_CC_FAT	anchoring junction	5	1.30E-02	9.50E-02
	GOTERM_BP_FAT	response to drug	5	3.60E-02	3.90E-01

	GOTERM_CC_FAT	cell junction	7	5.40E-02	2.60E-01
	SP_PIR_KEYWORDS	golgi apparatus	5	2.50E-01	7.60E-01
Annotation Cluster 13	Enrichment Score: 1.92				
	SP_PIR_KEYWORDS	zinc finger	5	4.30E-03	5.80E-02
	GOTERM_BP_FAT	protein complex assembly	9	8.50E-03	1.70E-01
	GOTERM_BP_FAT	protein complex biogenesis	9	8.50E-03	1.70E-01
	GOTERM_BP_FAT	macromolecular complex subunit organization	10	2.10E-02	2.80E-01
	GOTERM_BP_FAT	macromolecular complex assembly	9	3.70E-02	4.00E-01
Annotation Cluster 14	Enrichment Score:1.78				
	GOTERM_CC_FAT	cell projection	12	9.20E-04	1.40E-02
	GOTERM_CC_FAT	cell soma	5	1.20E-02	9.10E-02
	GOTERM_CC_FAT	neuron projection	6	3.30E-02	1.70E-01
	GOTERM_CC_FAT	dendrite	3	2.10E-01	6.00E-01
Annotation Cluster 15	Enrichment Score: 1.78				
	GOTERM_BP_FAT	response to organic substance	14	2.20E-04	1.80E-02
	GOTERM_BP_FAT	regulation blood vessel size	5	2.40E-04	1.80E-02
	GOTERM_BP_FAT	regulation of tuve size	5	2.40E-04	1.80E-02
	GOTERM_BP_FAT	vascular process in circulation system process	5	3.40E-04	2.30E-02
	GOTERM_CC_FAT	membrane raft	6	9.00E-04	1.50E-02
	GOTERM_BP_FAT	blood circulation	6	4.40E-03	1.20E-01
	GOTERM_BP_FAT	circulatory system process	6	4.40E-03	1.20E-01
	GOTERM_BP_FAT	negative regulation of endothelial cell proliferation	3	1.20E-01	1.20E-01
	GOTERM_BP_FAT	negative regulation of cell proliferation	8	4.70E-03	4.70E-03
	UP_SEQ_FEATURE	lipid oiety-binding region:S-palmitoyl cysteine	5	8.00E-03	6.10E-01
	GOTERM_BP_FAT	vasodilation	3	9.80E-03	1.90E-01

	SP_PIR_KEYWORDS	palmitate	5	1.40E-02	1.10E-01
	GOTERM_BP_FAT	regulation of endothelial cell proliferation	3	1.50E-02	3.10E-01
	GOTERM_BP_FAT	response to steroid hormone stimulus	5	2.50E-02	2.30E-01
	GOTERM_CC_FAT	caveola	3	3.00E-02	1.70E-01
	KEGG_PATHWAY	Focal adhesion adhesion	6	3.70E-02	4.10E-01
	GOTERM_BP_FAT	regulation of cellular component bogenesis	4	4.90E-02	4.40E-01
	GOTERM_BP_FAT	ditri-valent inorganic cation homeostasis	5	4.90E-02	4.40E-01
	GOTERM_BP_FAT	response to hormone stimulus	6	6.10E-02	5.00E-01
	GOTERM_BP_FAT	response to enogenous stimulus	6	8.40E-02	5.80E-01
	GOTERM_BP_FAT	positive regulation of signal transduction	5	9.10E-02	5.90E-01
	GOTERM_BP_FAT	regulation of system process	5	1.00E-01	6.20E-01
	GOTERM_BP_FAT	regulation of blood ressure	3	1.10E-01	6.50E-01
	GOTERM_BP_FAT	positive regulation of cell communication regulation	5	1.20E-01	6.70E-01
	GOTERM_BP_FAT	response to estrogen stimulus	3	1.20E-01	6.70E-01
	GOTERM_BP_FAT	second-messenger-mediated signaling	3	3.90E-01	9.50E-01
	GOTERM_MF_FAT	identical protein binding	5	4.90E-01	9.90E-01
	KEGG_PATHWAY	Neuroactive ligand-receptor interaction	3	6.90E-01	9.90E-01
Annotation Cluster 16	Enrichment Score:1.76				
	GOTERM_BP_FAT	protein kinase cascade	9	1.30E-03	5.70E-02
	GOTERM_BP_FAT	activation of NF-kappaB-inducing kinase activity	3	2.50E-03	9.80E-02
	GOTERM_BP_FAT	positive regulation of catalytic activity	10	2.90E-03	1.10E-01

	GOTERM_BP_FAT	regulation of protein kinase actiovity	8	3.70E-03	9.30E-02
	GOTERM_BP_FAT	regulation of kinase activity	8	4.50E-03	1.20E-01
	GOTERM_BP_FAT	regulation of transferase activity	8	5.60E-03	1.30E-01
	GOTERM_BP_FAT	I-kappaB kinase/NF-kappaB cascade	4	5.70E-03	1.30E-01
	GOTERM_BP_FAT	positive regulation of molecular function	10	6.40E-03	1.50E-01
	GOTERM_BP_FAT	regulation of phosphorus metabolic procession	9	6.70E-03	1.50E-01
	GOTERM_BP_FAT	regulation of phosphate metabolic	9	6.70E-03	1.80E-01
	GOTERM_BP_FAT	negative regulation of signal transduction	6	8.90E-03	9.30E-03
	GOTERM_BP_FAT	positive regulation of protein kinase activity	6	1.90E-01	1.80E-01
	GOTERM_BP_FAT	positive regulation of kinase activity	6	1.10E-02	1.40E-01
	GOTERM_BP_FAT	intracellular signaling cascade	15	1.20E-02	2.10E-01
	GOTERM_BP_FAT	positive regulaton of transferase activity	6	1.20E-02	2.20E-01
	GOTERM_BP_FAT	negative regulation of cell communication	6	1.40E-02	2.10E-01
	GOTERM_BP_FAT	regulation of phosphorylation	8	1.80E-02	2.60E-01
	GOTERM_BP_FAT	activation of protein kinase activity	4	2.80E-02	4.70E-02
	GOTERM_BP_FAT	positive regulation of response to stimulus	5	3.30E-01	4.40E-01
	SP_PIR_KEYWORDS	calcium binding	3	7.30E-02	3.80E-01
	GOTERM_BP_FAT	positive regulation of immune system process	4	7.90E-01	7.30E-01
	GOTERM_BP_FAT	regulation of MAP kinase activity	3	2.00E-01	6.20E-01
	GOTERM_CC_FAT	external side of plasma membrane	3	2.20E-01	1.60E-01
	SP_PIR_KEYWORDS	calcium	5	4.60E-01	9.10E-01

	GOTERM_MF_FAT	calcium ion binding	4	9.00E-01	1.00E+00
Annotation Cluster 17	Enrichment Score:1.63				
	GOTERM_BP_FAT	response to hypoxia	8	1.20E-05	1.70E-03
	GOTERM_BP_FAT	response to oxygen levels	8	1.60E-05	2.10E-03
	GOTERM_BP_FAT	positive regulation of cell adhesion	4	5.00E-03	1.20E-01
	GOTERM_BP_FAT	regulation of cell-matrix adhesion	3	1.00E-02	1.90E-01
	GOTERM_BP_FAT	positive regulation of cell-sbustrate adhesion	3	1.10E-02	1.50E-02
	GOTERM_BP_FAT	negative regulation of muticellular organismal process	5	2.00E-01	2.30E-01
	GOTERM_BP_FAT	regulation of transforming growth factor beta receptor sinaling	3	2.10E-02	2.80E-01
	GOTERM_BP_FAT	positive regulaton of developmental process	6	2.20E-02	3.10E-01
	GOTERM_BP_FAT	response to steroid hormone stimulus	5	2.50E-02	2.90E-01
	GOTERM_BP_FAT	regulation of cell-substrate adhesion	3	2.90E-02	3.40E-01
	GOTERM_BP_FAT	negative regulation of cell migration	3	4.30E-02	4.30E-01
	GOTERM_BP_FAT	regulation of cell adhesion	4	4.50E-02	4.20E-01
	GOTERM_BP_FAT	negative regulation of locomotion	3	4.80E-02	4.40E-01
	GOTERM_BP_FAT	negative regulation of cell motion	3	5.10E-02	4.50E-01
	GOTERM_BP_FAT	regulation of cell migration	4	7.40E-02	5.50E-01
	GOTERM_BP_FAT	positive regulation of signal transduciton	5	9.10E-02	5.90E-01
	GOTERM_BP_FAT	regulation of	4	9.90E-02	6.20E-0

		locomotion			1
	GOTERM_BP_FAT	regulation of cell motion	4	1.00E-01	6.20E-0 1
	GOTERM_BP_FAT	positive regulation of cell cmommunication	5	1.20E-01	6.70E-0 1
	GOTERM_BP_FAT	blood vessel morphgenesis	4	1.20E-01	6.70E-0 1
	GOTERM_BP_FAT	blood vessel development	4	1.70E-01	7.50E-0 1
	GOTERM_BP_FAT	vasculature development	4	1.80E-01	7.60E-0 1
	GOTERM_BP_FAT	negative regulation of cell differentiation	3	3.50E-01	9.30E-0 1
Annotation Cluster 18	Enrichment Score:1.48				
	GOTERM_BP_FAT	positive regulation of cytokine production	5	1.80E-03	7.30E-02
	GOTERM_BP_FAT	regulation of of cytokine production	6	3.90E-03	1.10E-01
	GOTERM_BP_FAT	positive regulation response to stimulus	5	9.20E-01	4.70E-02
	GOTERM_BP_FAT	posttranscriptional regulation of gene expression	3	3.40E-01	4.40E-01
	GOTERM_BP_FAT	immune response	6	3.70E-01	9.40E-01
Annotation Cluster 19	Enrichment Score:1.42				
	GOTERM_BP_FAT	positive regulation of developmental process	6	2.20E-02	4.70E-02
	GOTERM_BP_FAT	positive regulation of response to stimulus	5	2.90E-01	4.40E-01
	GOTERM_BP_FAT	positive regulation of response to external timulus	3	5.30E-02	4.50E-01
Annotation Cluster 20	Enrichment Score:1.38				
	GOTERM_CC_FAT	apical plasma membrane	4	3.30E-02	1.70E-01

	GOTERM_BP_FAT	vesicle organization	3	3.60E-02	3.90E-01
	KEGG_PATHWAY	Focal adhesion	6	3.70E-02	4.10E-01
	GOTERM_CC_FAT	apical part of cell	4	6.80E-02	3.10E-01
